# Author Correction: Loss of Fsr quorum sensing promotes biofilm formation and worsens outcomes in enterococcal infective endocarditis

**DOI:** 10.1038/s41467-026-78152-1

**Published:** 2026-09-25

**Authors:** Haris Antypas, Verena Schmidtchen, Willy Isao Staiger, LI Yanhong, Rachel Jing Wen Tan, Kenneth Kok Fei NG, Cheryl Jia Yi Neo, Shalome Meera Radhesh, Frederick Reinhart Tanoto, Ronni Anderson Gonçalves da Silva, Cristina Colomer-Winter, Sara Doina Schütz, Joachim Kloehn, Logeshwari Muthualagu Natarajan, Caroline Manzano, Jun Jie Wong, Kevin Pethe, Barbara Hasse, Silvio Daniel Brugger, Siu Ling Wong, Daria Van Tyne, Annelies S. Zinkernagel, Kimberly A. Kline

**Affiliations:** 1 Singapore Centre for Environmental Life Sciences Engineering, School of Biological Sciences, Nanyang Technological University, Singapore, Singapore; 2 Division of Infectious Diseases, University Hospital Zurich, Zurich, Switzerland; 3 Department of Medicine, University of Pittsburgh, Pittsburgh, PA USA; 4 School of Medicine, Tsinghua University, Beijing, China; 5 Singapore-MIT Alliance for Research and Technology Centre, Singapore, Singapore; 6 Department of Microbiology and Molecular Medicine, University of Geneva, Geneva, Switzerland; 7 Lee Kong Chian School of Medicine, Nanyang Technological University, Singapore, Singapore; 8 National Center for Infectious Diseases (NCID), Singapore, Singapore; 9 Tan Tock Seng Hospital, Singapore, Singapore

**Keywords:** Biofilms, Pathogens

Correction to: *Nature Communications* https://doi.org/10.1038/s41467-026-68366-8, published online 14 January 2026

In the version of this article initially published, in the Fig. 4f Source Data and associated figure panel, eight measurements in the the Δ*gelE* microcolony dataset were inadvertent duplicates. The Source Data and figure have been updated, while in the Fig. 4f legend, the number of Δ*gelE* microcolonies has been amended from 221 to 213. In the Supplementary Fig. S2a, S2e Source Data, the Δ*fsr* section contained biological replicate 2 twice due to a copy-paste error, while the true biological replicate 1 was absent, and the Source Data originally contained four instead of three WT and Δ*gelE* experiments. In the Supplementary Fig. 9 Source Data, labels for S9A and S9C were swapped; the label for panel S9G was also corrected from cylB to cylL. Minor spelling errors were corrected in the Figs. 5–7 Source Data labels. The Source Data, Fig. 4 image and legend are updated in the HTML and PDF versions of the article. For comparison, the original Fig. 4 is available as Supplementary Information accompanying this amendment.

## Supplementary information

Original Fig. 4

