# Supplementary material for: Loss of Fsr quorum sensing promotes biofilm formation and worsens outcomes in enterococcal infective endocarditis
Source: Nat Commun. 2026 Jan 14;17:1668. doi: 10.1038/s41467-026-68366-8 (PMC12909850; doi:10.1038/s41467-026-68366-8)
Supplement: Supplementary file 1 — Supplementary Information [file 41467_2026_68366_MOESM1_ESM.pdf]

SUPPLEMENTARY INFORMATION

Loss of Fsr quorum sensing promotes biofilm formation and worsens outcomes in enterococcal infective endocarditis

Haris ANTYPAS[1,*], Verena SCHMIDTCHEN[2], Willy Isao STAIGER[2], Yanhong LI[3,4], Rachel Jing Wen TAN[1], Kenneth Kok Fei NG[1], Cheryl Jia Yi NEO[1], Shalome Meera RADHESH[1], Frederick Reinhart TANOTO[1], Ronni Anderson Gonçalves DA SILVA[1,5], Cristina COLOMER-WINTER[6], Sara Doina SCHÜTZ[2], Joachim KLOEHN[6], Logeshwari MUTHUALAGU NATARAJAN[1], Caroline MANZANO[6], Jun Jie WONG[1], Kevin PETHE[1,5,7,8], Barbara HASSE[2], Silvio Daniel BRUGGER[2], Siu Ling WONG[7,9], Daria VAN TYNE[3], Annelies S. ZINKERNAGEL[2], Kimberly A. KLINE[1,5,6,*]

[1]Singapore Centre for Environmental Life Sciences Engineering, School of Biological Sciences, Nanyang Technological University, Singapore

[2]Division of Infectious Diseases, University Hospital Zurich, CH-8091 Zurich, Switzerland

[3]Department of Medicine, University of Pittsburgh, PA, USA

[4]School of Medicine, Tsinghua University, Beijing, China

[5]Singapore-MIT Alliance for Research and Technology Centre, Singapore

[6]Department of Microbiology and Molecular Medicine, University of Geneva, Switzerland

[7]Lee Kong Chian School of Medicine, Nanyang Technological University, Singapore

[8]National Center for Infectious Diseases (NCID), 308442, Singapore

[9] Tan Tock Seng Hospital, Singapore

[*]Correspondence: Haris.Antypas@ntu.edu.sg, Kimberly.Kline@unige.ch

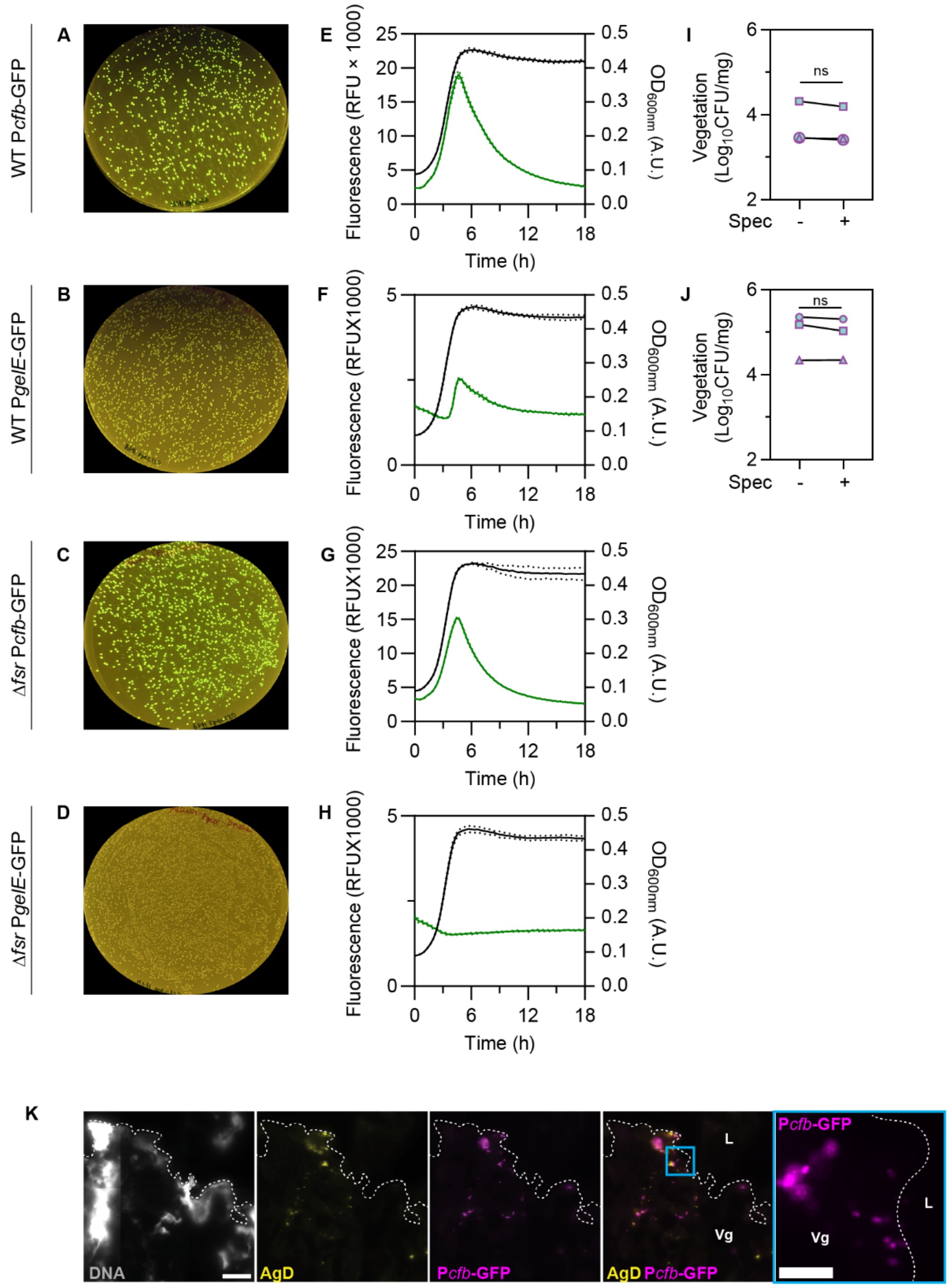

Fig. S1. Reporter plasmid P*gelE*-GFP emits fluorescence in response to QS activation *in vitro* and *in vivo*. A-D. *E. faecalis* OG1RF WT and Δ*fsr* were transformed with reporter plasmids P*cfb*-GFP (constitutive promoter) and P*gelE*-GFP (QS-regulated *gelE* promoter). On agar plates, exposure to blue light transilluminator resulted in fluorescence emission in all strains except from Δ*fsr* P*gelE*-GFP (D), showing that P*gelE*-GFP expression is Fsr-dependent. N = 1. **E-H.** Growth curves of WT P*cfb*-GFP (E), WT P*gelE*-GFP (F), Δ*fsr* P*cfb*-GFP (G), Δ*fsr* P*gelE*-GFP (H) in M1 supplemented with glucose (110mM) at 37°C under aerobic non-shaking conditions further validated the Fsr-dependent expression of P*gelE*-GFP. Green solid line shows the mean fluorescence (ex488nm/em522nm) and black solid line shows the OD_{600nm} from 3 technical replicates of a single representative biological replicate out of N = 3. Error

= SD represented as dotted line. **I-J.** CFU counts from vegetation homogenates (n = 3, N = 2) plated on BHI and BHI + spectinomycin agar to assess plasmid stability in WT P*cfb*-GFP (I) and WT P*gelE*-GFP (J) strains at 24 hpi. Lines connect CFU counts from the same homogenate sample. Significance was assessed with two-tailed paired t-test. The 3 different symbols correspond to 3 different animals. **K.** P*cfb*-GFP expression in WT bacteria remains stable independently of their distance from the vegetation (Vg) surface at 24 hpi. The blue inset highlights P*cfb*-GFP expression in bacteria located near the vegetation surface. Image was acquired with epifluorescence tiling microscopy and stained for DNA and *Enterococcus*-specific Group D *Streptococcus* antigen (AgD). L = lumen, dashed line = vegetation boundary. Representative image from n = 2, N = 1. Scale = 50 µm (main panel) and 5 µm (inset); ns = not significant ($p \geq 0.05$), n = animals per group, N = independent experiments, A.U. = arbitrary units, RFU = relative fluorescence units. Source data are provided as a Source Data file.

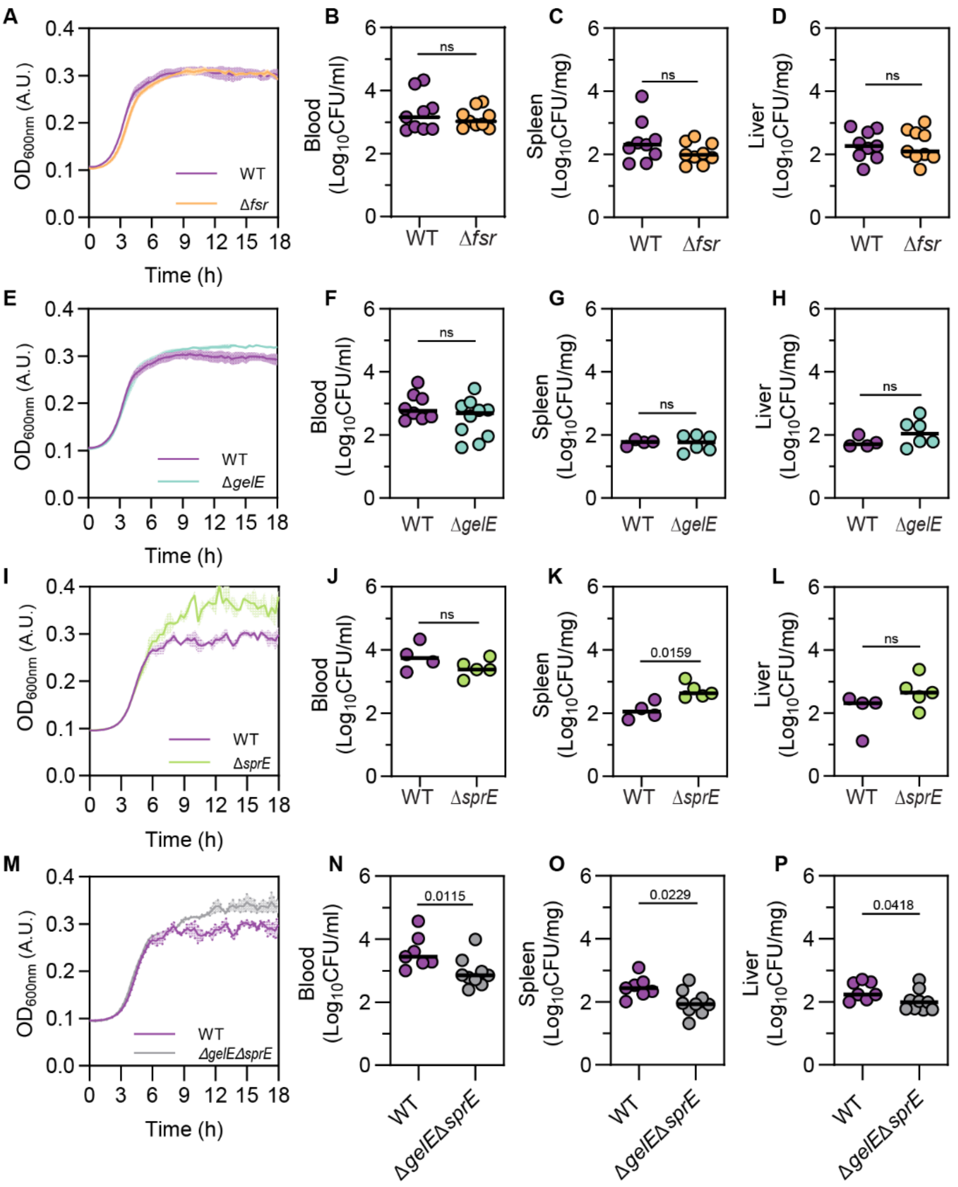

Fig. S2. Growth and systemic spread of *E. faecalis* in the absence of Fsr QS system, *gelE*, and *sprE* in IE at 72 hpi. A, E, I, M. Growth curves in BHIS at 37°C under aerobic conditions without shaking. Error = SEM represented as shaded region. N = 3 for (A) and (E), N = 1 for (I) and (M). **B-D, F-H, J-L, N-P.** Median of blood, liver, and spleen CFU at 72 hpi. n = 9 from N = 2 for (B-D), n = 8 (WT) & n = 10 (Δ*gelE*) from N = 2 for (F), n = 4 (WT) & n = 6 (Δ*gelE*) from N = 1 for (G-H), n = 4 (WT) & n = 5 (Δ*sprE*) from N = 1 for (J-L), n = 7 (WT) & n = 9 (Δ*gelE*Δ*sprE*) from N = 2 for (N-P). Statistical significance was assessed with a two-tailed Mann-Whitney test; ns = not significant ($p \geq 0.05$), n = animals per group, N = independent experiments, A.U. = arbitrary units. Exact p-values are reported in the figure. Source data are provided as a Source Data file.

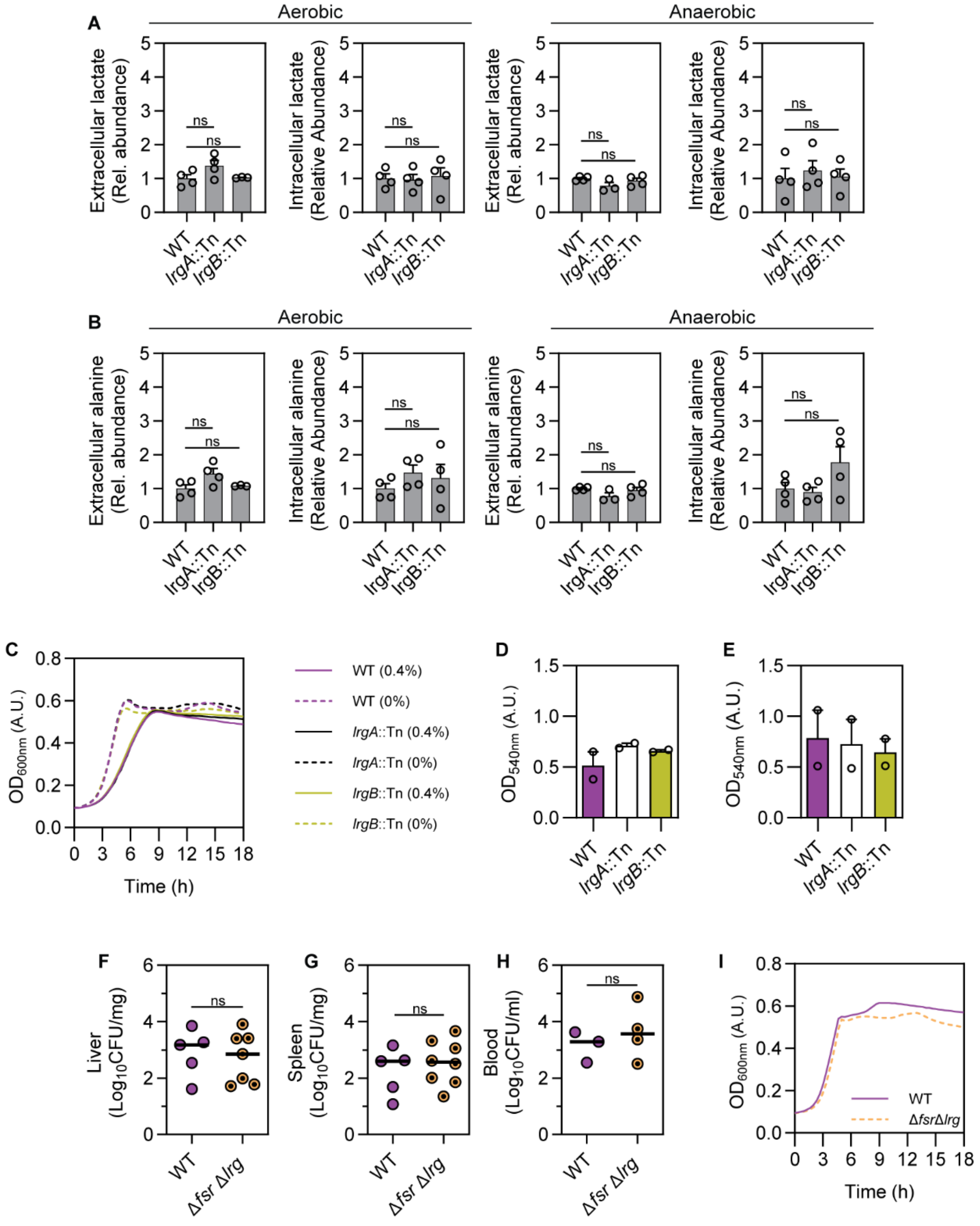

Fig. S3. *lrgAB* does not contribute to pyruvate conversion to lactate or alanine, Triton X-100 sensitivity, or biofilm formation. A-B. Relative abundance of extracellular and intracellular lactate (A) or alanine (B) was measured in *E. faecalis* WT, *lrgA*::Tn, and *lrgB*::Tn cultures grown in M1 supplemented with 10 mM pyruvate under aerobic and anaerobic conditions using GC-MS. Mean and SEM for N = 4, except extracellular pyruvate in aerobic *lrgB*::Tn and anaerobic *lrgA*::Tn cultures (N = 3). Statistical analysis was performed using one-way ANOVA followed by Tukey's multiple comparisons test. **C.** Growth curves of *E. faecalis* strains in BHI with 0.4 or 0 % Triton X-100 at 37°C under aerobic conditions without shaking. Data represents the mean of 3 technical replicates from a representative experiment out of N = 3. **D-E.** Biofilm formation at 24 h on tissue culture-treated (D) and uncoated polystyrene (E) 96-well plates assessed with the crystal violet assay. Mean of N = 2 is shown. **F-H.** Liver, spleen, and blood CFU at 72 hpi. Data represent the median from n = 5 (WT) & n = 8 (Δ*fsr* Δ*lrg*) for

liver and spleen, and n = 3 (WT) & n = 4 (Δ*fsr* Δ*lrg*) for blood, all from *N* = 1. Missing blood samples were due to clotting. Statistical significance was assessed with a two-tailed Mann-Whitney test. **I.** Growth curves in BHI at 37°C under aerobic conditions without shaking. Mean OD600nm of 3 technical replicates is shown. N = 1; ns = not significant ($p \geq 0.05$), n = animals per group, N = independent experiments, A.U. = arbitrary units. Source data are provided as a Source Data file.

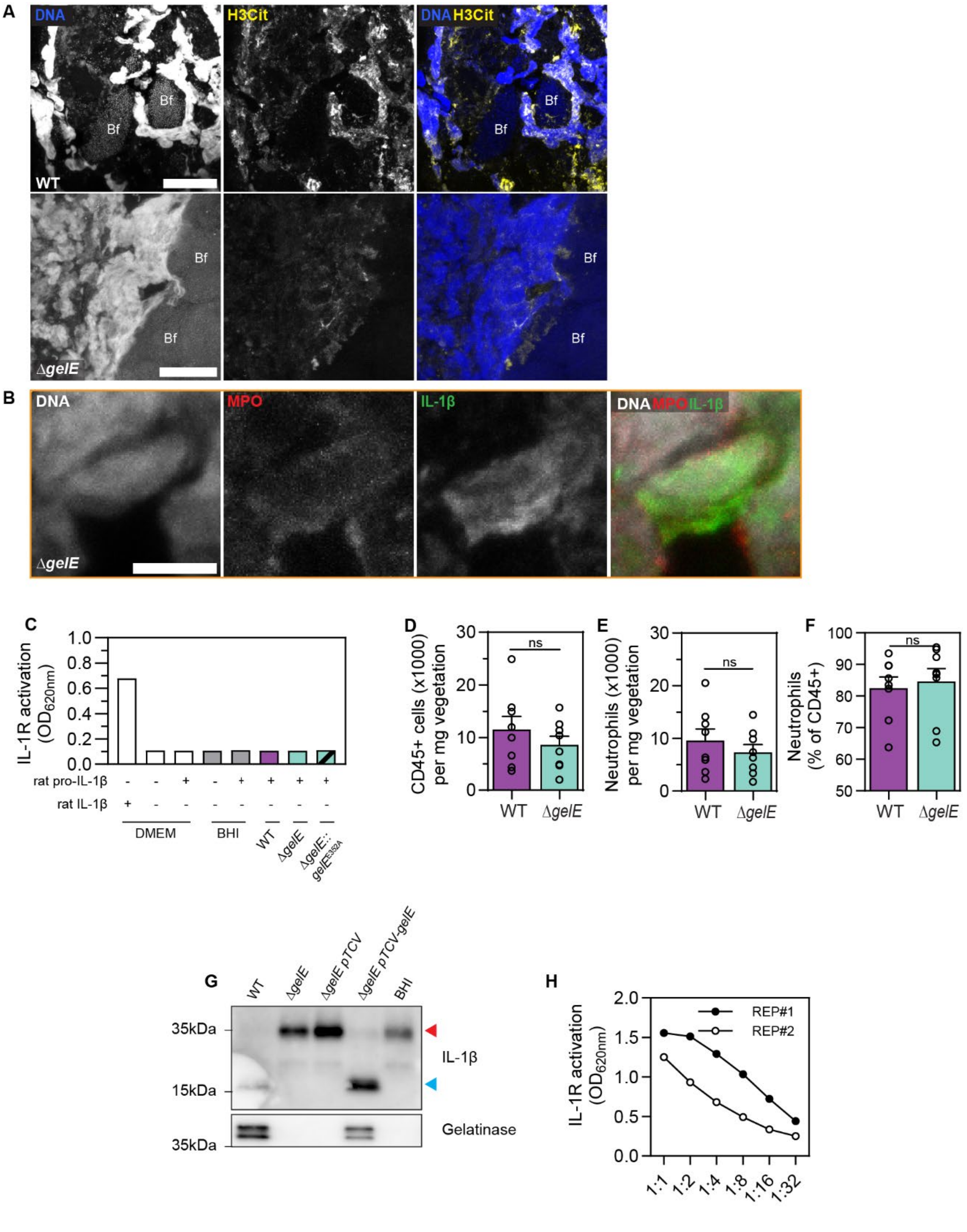

Fig. S4. **Neutrophil recruitment, NETosis, and gelatinase-dependent human pro-IL-1β activation in IE.** **A.** Neutrophils undergoing NETosis at the interface with biofilm, evidenced by their decondensed nuclei colocalizing with citrullinated histone H3 (H3Cit) in WT- and Δ*gelE*-infected vegetations at 72 hpi. Representative Z-projection captured with LSCM from n = 3, N = 1 is shown. DNA is stained with DAPI. Bf = biofilm, Scale = 20 µm. **B**. Inset (orange) from Fig. 6A (Δ*gelE* panel) showing IL-1β colocalizing with a decondensed nucleus of an incoming neutrophil. Colocalization with myeloperoxidase (MPO) indicates that the nucleus belongs to a neutrophil. Scale = 5 µm. **C.** HEK-Blue IL-1R reporter cells

incubated in the presence of supernatants harvested from OG1RF WT and Δ*gelE*::*gelE*^{E352A} cultures with rat pro-IL-1β at 6 h. Cell activation was assessed by spectrophotometric measurement of reporter cell supernatants incubated with a chromogenic substrate. Stimulation of cells with mature rat IL-1β directly added to the reporter cells was used as a positive control. N = 1. **D-F.** Leukocyte (CD45+) (D) and neutrophil (E) absolute quantification, and neutrophil relative quantification (F) at 72 hpi vegetations using flow cytometry. Neutrophils (% of CD45+ cells) were determined based on the number of CD45+ RP-1+ events of the total CD45+ events. Mean with SEM is shown from n = 8, N = 2. Statistical significance was assessed with a two-tailed t-test. **G.** Complementation of Δ*gelE* with pTCV-P*tet-gelE* restored secretion of gelatinase and cleavage of pro-IL-1β. Human pro-IL-1β was incubated in BHI with OG1RF WT, Δ*gelE*, Δ*gelE* pTCV-*gelE*, or Δ*gelE* pTCV (empty vector) at 37°C and supernatants were collected at 18 h. Western blotting detected gelatinase and pro-IL-1β cleavage to a 17 kDa fragment. BHI alone served as negative control. Red arrowhead = pro-IL-1β, blue arrowhead = mature IL-1β. **H.** Activation of HEK-Blue IL-1R reporter cells in the presence of 2-fold serial dilutions of supernatants harvested from OG1RF WT incubated with pro-IL-1β for 18 h. N = 2; ns = not significant ($p \geq 0.05$), n = animals per group, N = independent experiments. Source data are provided as a Source Data file except for Fig. S4G, which is provided at the end of this Supplementary Information file.

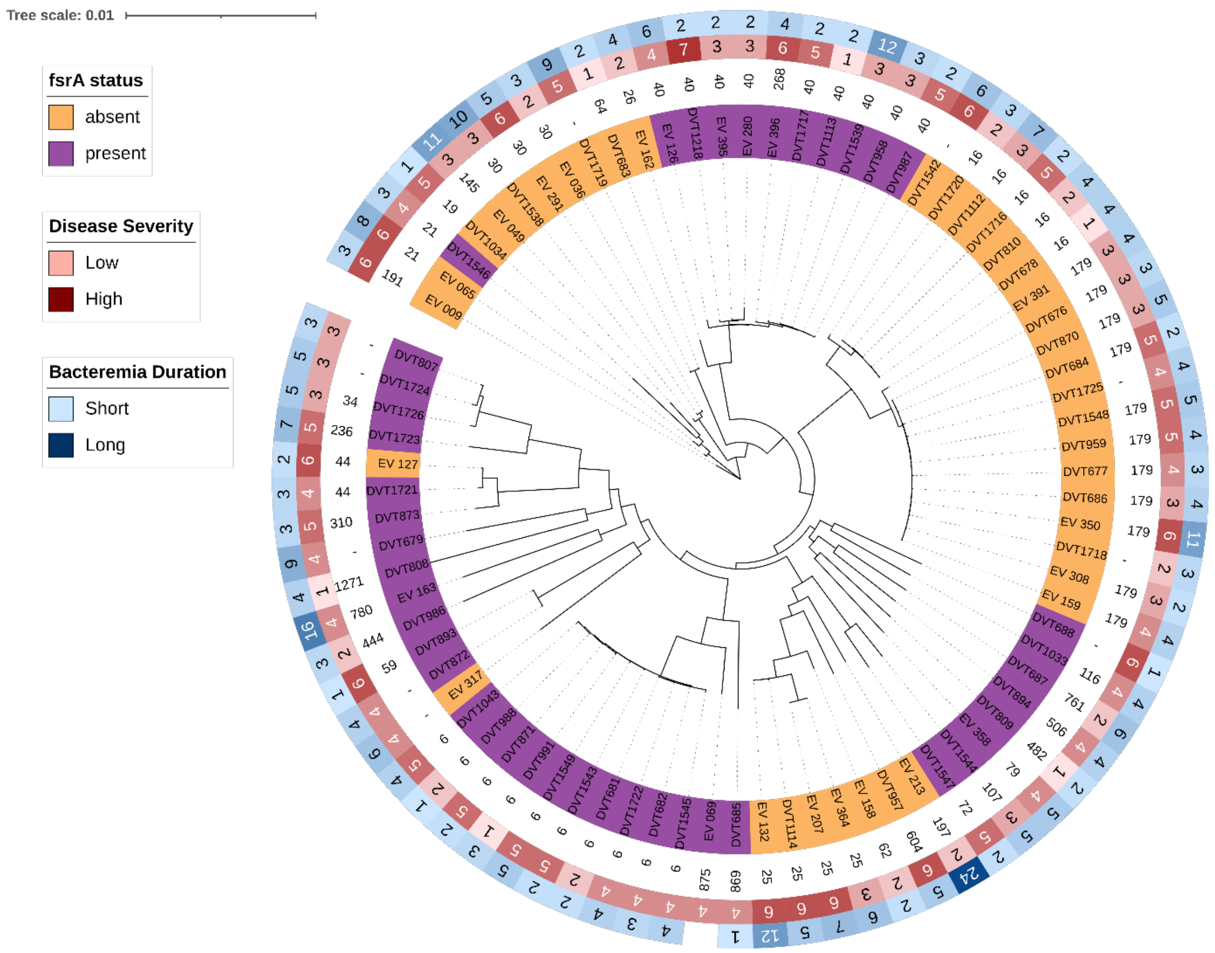

Fig. S5. Phylogenetic tree of *E. faecalis* IE isolates. The innermost ring indicates *fsrA* presence (orange = absent; purple = present) and isolate ID. Strains from the Pittsburgh cohort are labelled as DVT####, and those from the ENVALVE cohort as EV_###. The second ring shows sequence type (ST), the third ring shows disease severity score, and the outermost ring displays bacteremia duration in days.

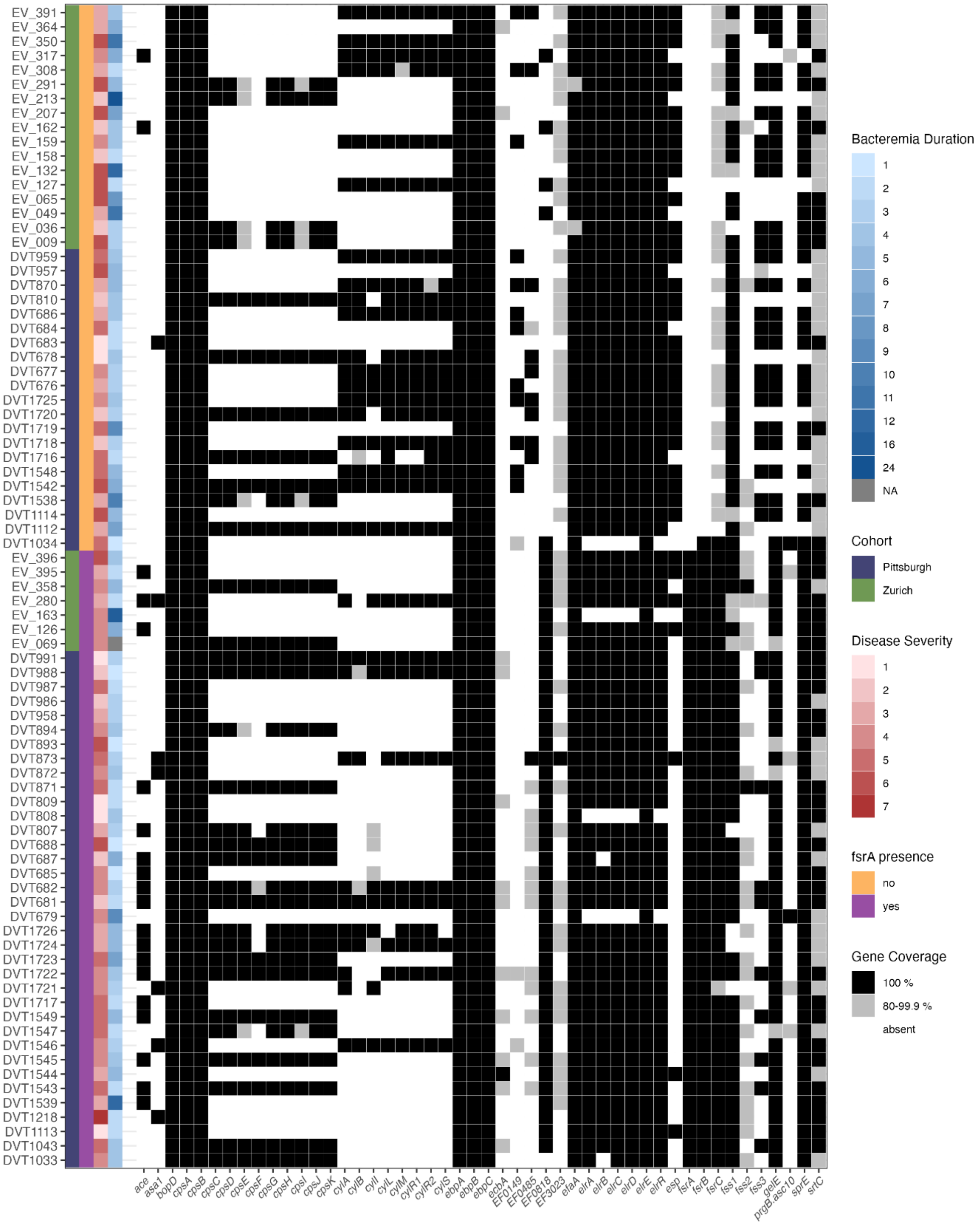

Fig. S6. Heatmap showing the presence of virulence factors (X axis) across clinical isolates (Y axis) included in this study. Gene presence was assessed with whole genome sequencing. Strains from the Pittsburgh cohort are labelled as DVT####, and those from the ENVALVE cohort as EV_###. The first four columns (from left to right) indicate cohort origin, *fsrA* presence, disease severity, and duration of bacteremia. Corresponding color codes are shown on the right side of the heatmap. The remaining columns represent individual virulence factor genes, with color coding denoting absence (white), presence (black), or partial presence (grey; 80–99.9% gene coverage).

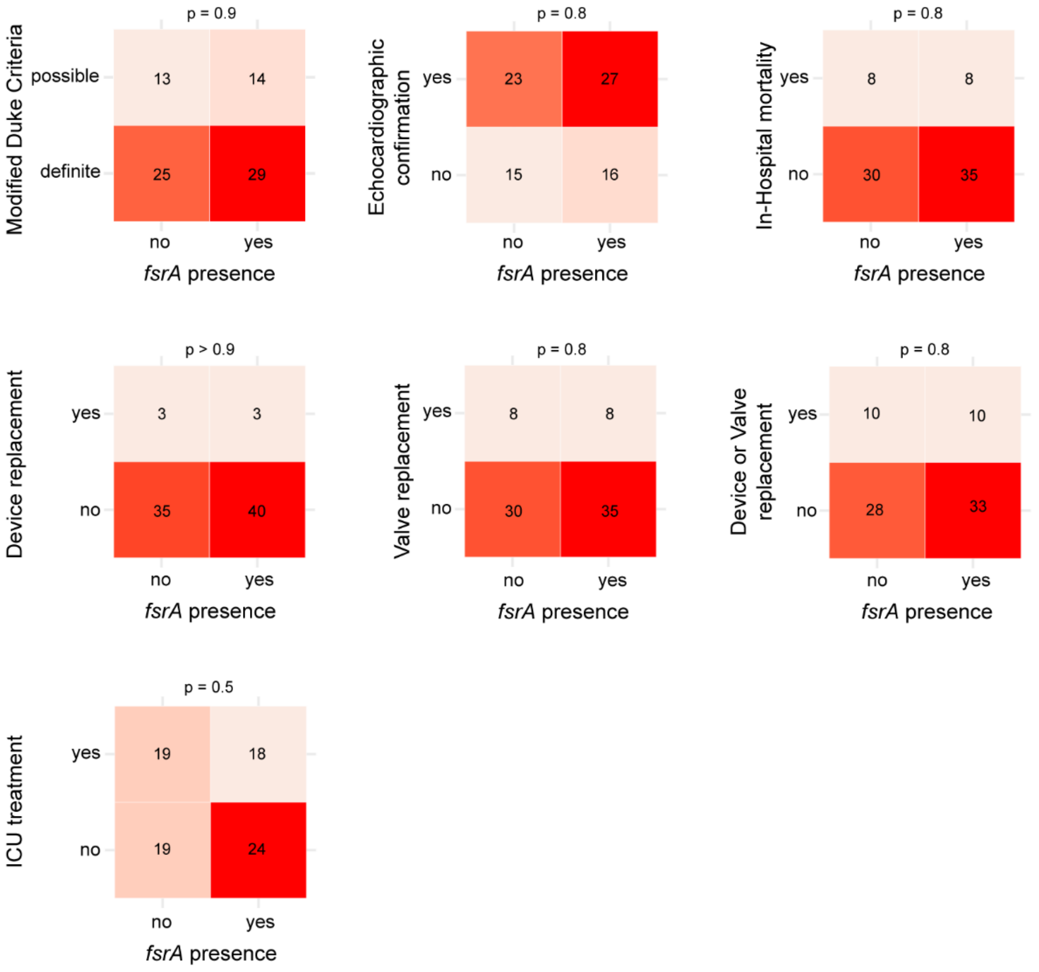

Fig. S7. **Relationship between *fsr* presence and clinical parameters of IE.** Contingency tables of the association of *fsrA* presence with different clinical parameters are shown. Statistical significance was assessed by Fisher's exact test. Exact p-values are reported in the figure.

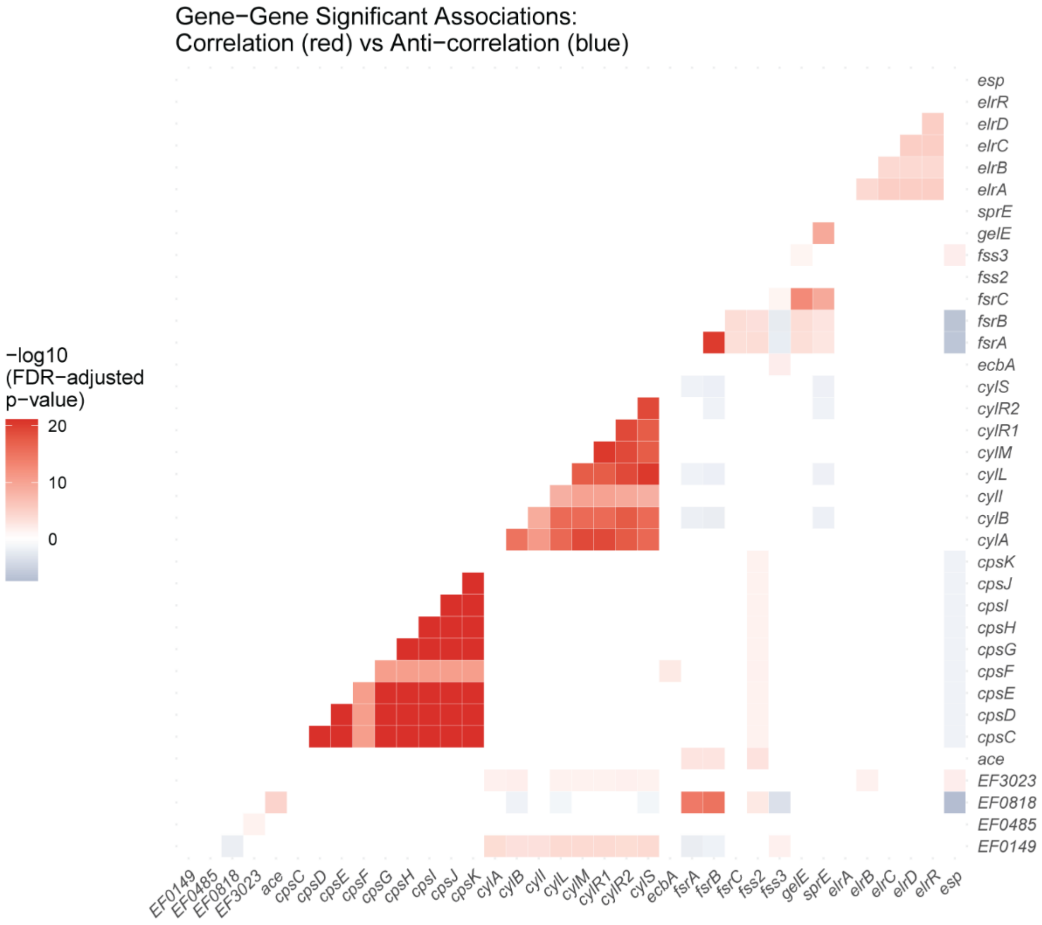

Fig. S8. **Gene-gene correlation analysis among *E. faecalis* virulence factors.** Heatmap showing significant gene-gene associations identified across the IE clinical isolates used in this study. Correlations are displayed in red and anti-correlations in blue, with significance plotted as –log10(FDR-adjusted p-value).

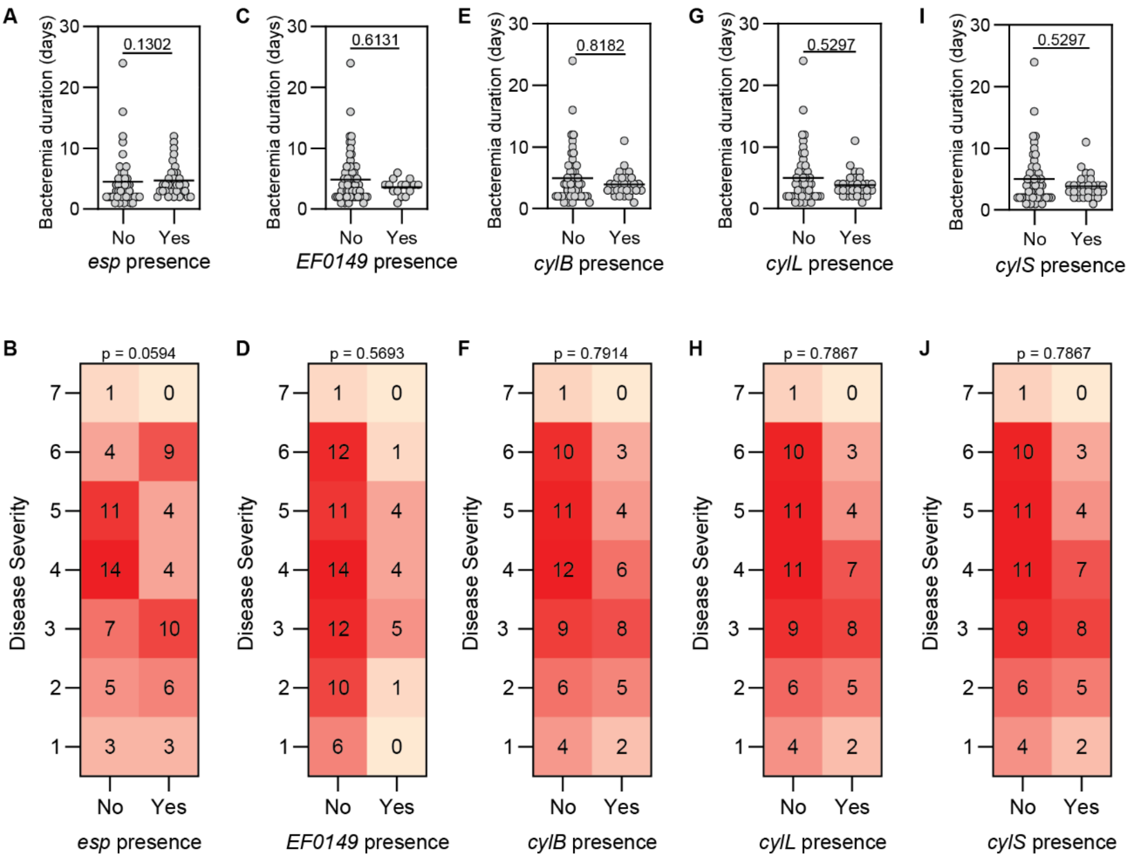

Fig. S9. Virulence factors *esp*, *EF0149*, *cylB*, *cylL*, *cylS* anticorrelating with *fsrA* presence are not associated with prolonged bacteremia and high disease severity score in IE patients. A, C, E, G, I. Bacteremia duration of IE patients in relation to different virulence factors presence. Median is shown. Statistical significance was determined with a Mann-Whitney test with continuity correction. **B, D, F, H, J.** Cumulative disease severity score (Y-axis) of IE patients infected in relation to different virulence factor presence (X-axis). Frequency (n) for each disease score is shown within each heatmap box. Statistical significance was determined with Fisher's exact test; Exact p-values are reported in the figure. Source data are provided as a Source Data file.

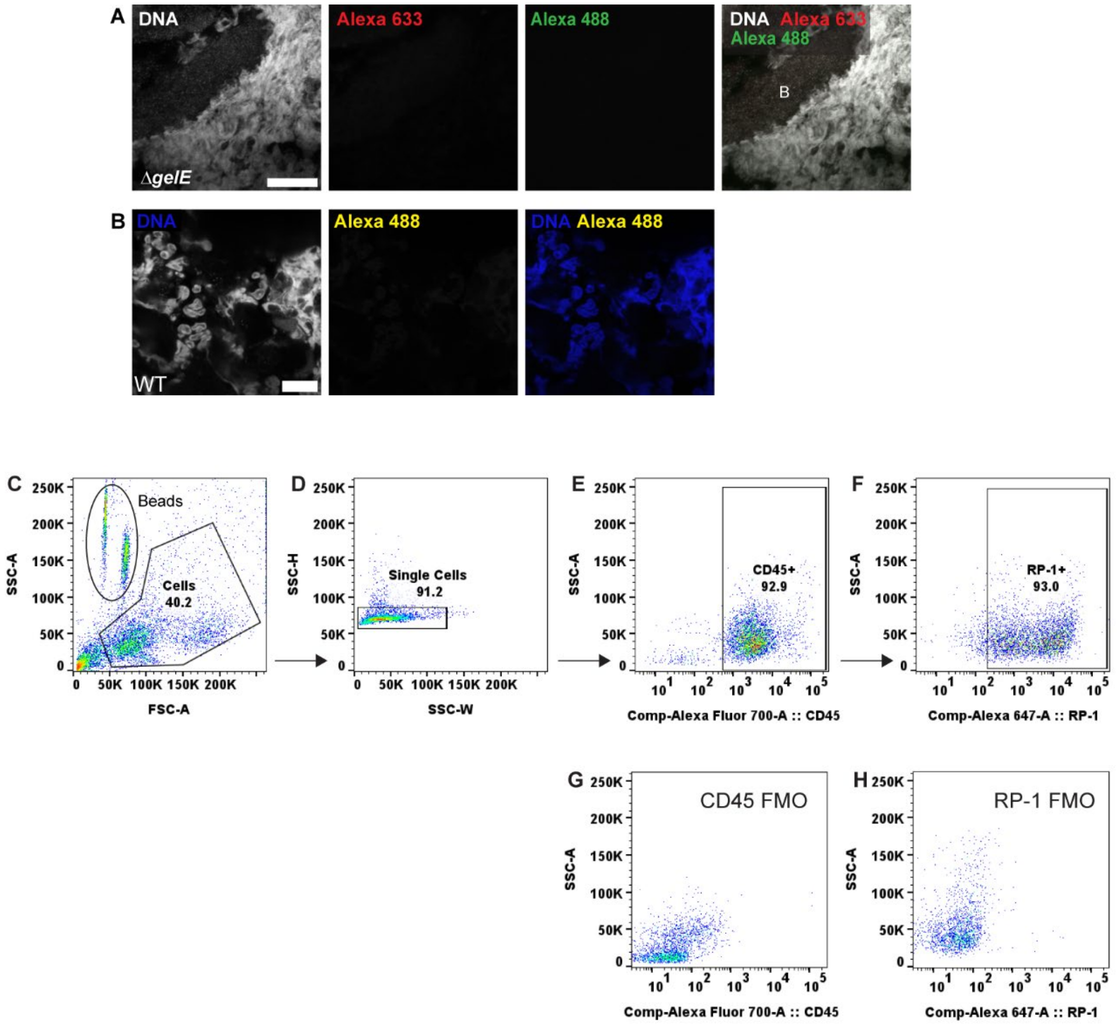

Fig. S10. Secondary antibody only controls and flow cytometry gating strategy. A. Secondary antibody only control performed on a tissue section consecutive to the section shown in Fig. 6A (Δ*gelE* panel). Z-projection of images captured with LSCM is shown, stained for DNA, goat anti-rabbit IgG Alexa 488, and goat anti-mouse IgG1 Alexa 633. B = biofilm, scale = 20 µm. **B.** Z-projections of images captured from WT-infected vegetations at 72 hpi captured with LSCM, stained for DNA and goat anti-rabbit IgG Alexa 488. Representative image of neutrophils undergoing NETosis from N=3 is shown. Scale = 20 µm. **C-H**. Flow cytometry gating to identify rat neutrophils (CD45+RP-1+) in vegetations was performed as follows: total cell population (C) was selected based on forward and side scatter (FSC/SSC); in this plot, the two bead populations used for absolute quantification can also be distinguished. Single cells (D) were identified by SSC-W vs SSC-H, CD45+ cells (E) were gated based on fluorescence from anti-CD45 antibody staining, and RP-1+ (F) cells were subsequently selected from the CD45+ population based on fluorescence from anti–RP-1 antibody staining. Fluorescence minus one (FMO) controls are shown for CD45 (G) and RP-1 (H) to define positive gating boundaries; N = independent experiments.

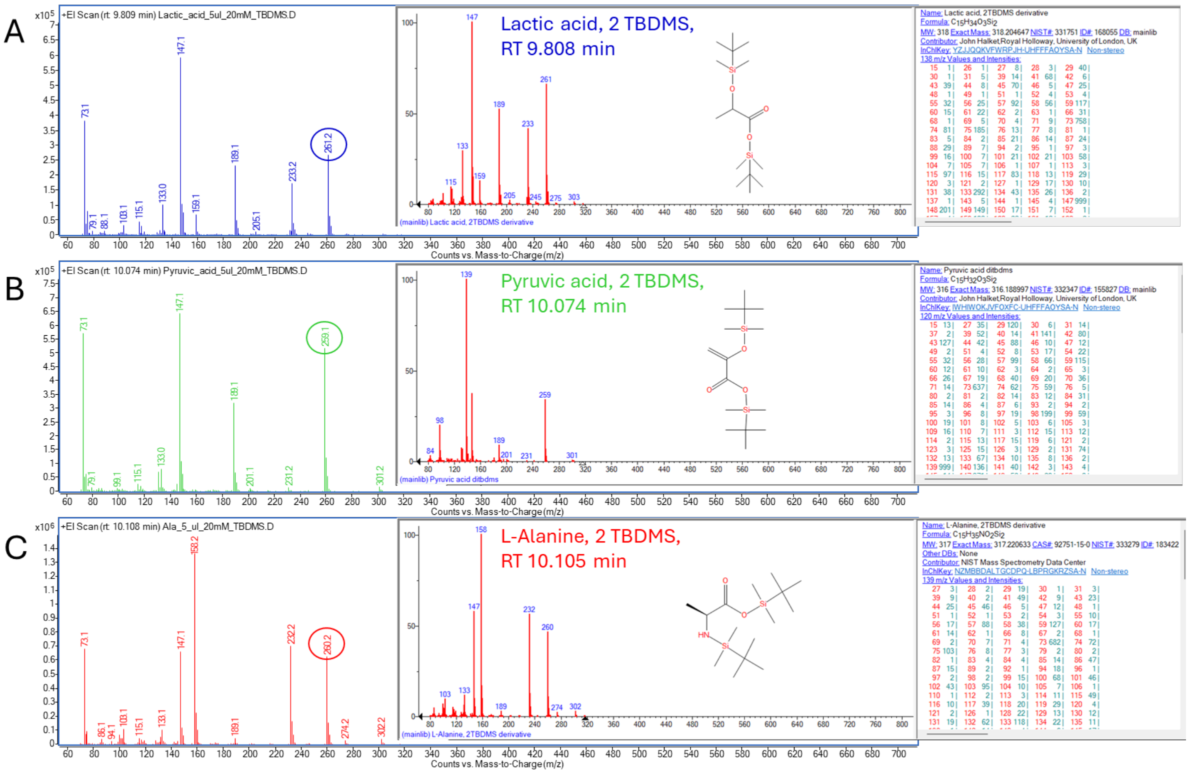

Fig. S11. Analysis of relevant Standards. A-C. Ion spectra of authentic standards of lactic acid (A), pyruvic acid (B) and L-alanine (C), following derivatization with N-tert-butyldimethylsilyl-N-methyl trifluoroacetamide (MTBSTFA) containing 1% tert-butyldimethylchlorosilane (TBDMCS) and analysis by gas chromatography-mass spectrometry (GC-MS) in EI mode (70 eV), acquiring data in Scan mode (m/z 70-700). Screenshots of the corresponding National Institute of Standards and Technology (NIST) library spectra, as well as the retention times (RTs) are given. The ion used for quantification of the respective metabolite is highlighted by a circle and corresponds for each 2TBDMS metabolite to the [M-57]+ ion. Note that metabolite quantifications were also performed using another suitable ion for each metabolite (lactic acid: m/z 233, pyruvic acid: m/z 189, L-alanine: m/z 232), giving comparable results.

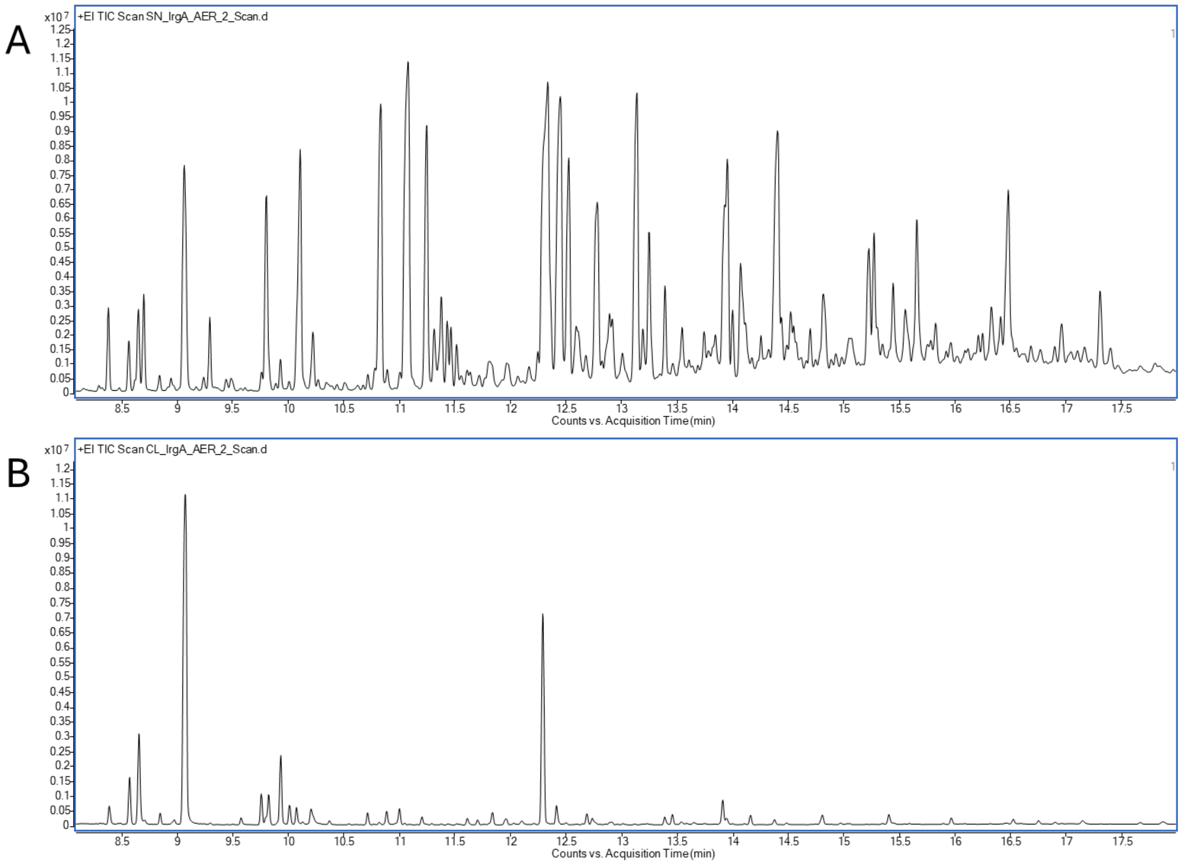

Fig. S12. Total Ion Chromatograms. A-B. Representative Total Ion Chromatograms (TICs) from a supernatant sample (A) and a cell extract (B), following metabolite extraction (N = 4), derivatization with N-tert-butyldimethylsilyl-N-methyl trifluoroacetamide (MTBSTFA) containing 1% tert-butyldimethylchlorosilane (TBDMCS) and analysis by gas chromatography-mass spectrometry (GC-MS) in EI mode (70 eV), acquiring data in Scan mode (m/z 70-700). The samples displayed here are both from the *lrgA*::Tn strain cultured under aerobic conditions, replicate 2.

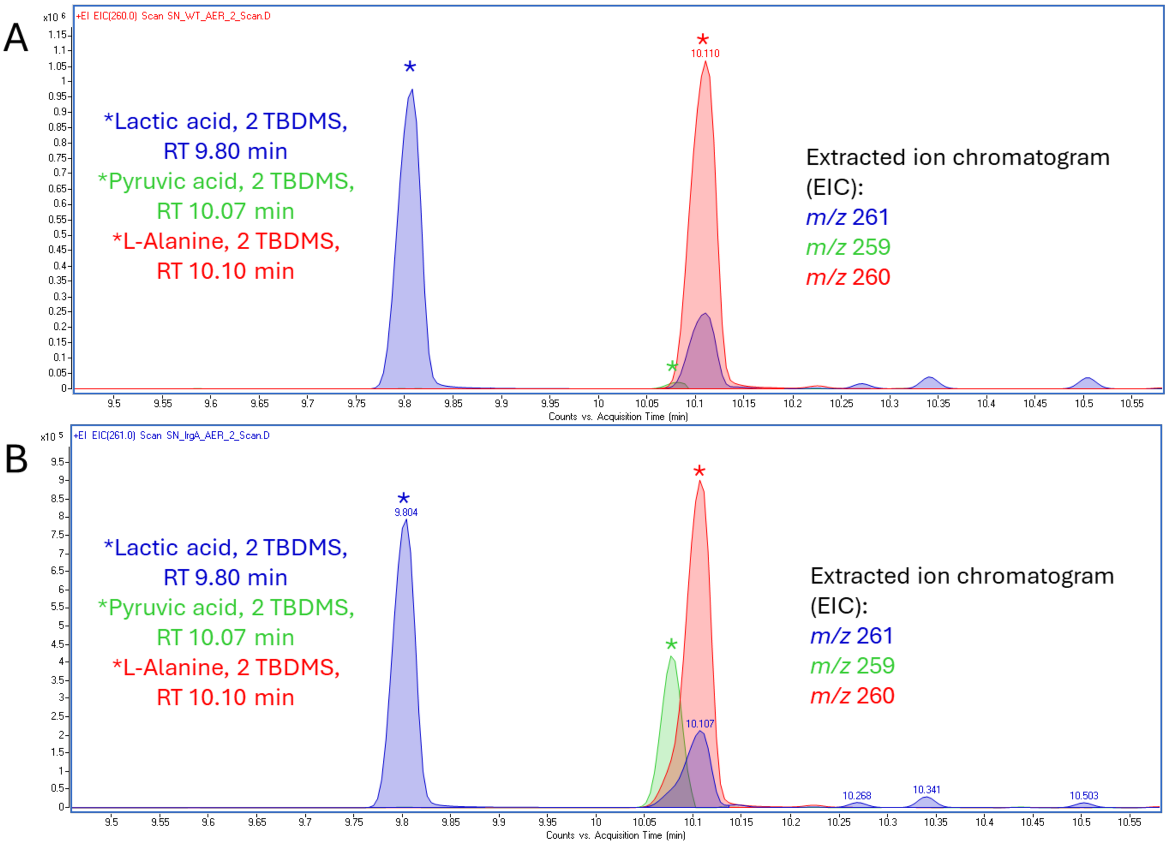

Fig. S13. Extracted Ion Chromatograms. Representative Extracted Ion Chromatograms (EICs) from 2 samples: Supernatant of WT (A) and *lrgA*::Tn (B) cultured under aerobic conditions (replicate No 2 for both, N = 4). The extracted ions are m/z 261 (blue), 259 (green) and 260 (red), corresponding to the [M-57]+ ion for lactic acid, pyruvic acid and L-alanine respectively (all 2TBDMS derivatives). Increased pyruvic acid levels in the *lrgA*::Tn compared to the WT supernatant sample are apparent and were quantified in this study.

Table S1. MIC of ampicillin for *E. faecalis* OG1RF WT, *lrgA*::Tn, and *lrgB*::Tn strains

	MIC (μg/ml)		
	**Rep#1**	**Rep#2**	**Rep#3**
**WT**	0.5	1	2
***lrgA*::Tn**	0.5	1	2
***lrgB*::Tn**	0.5	1	2

Table S2. MIC of gentamicin for *E. faecalis* OG1RF WT and Δ*fsr* strains

	MIC (μg/ml)		
	**Rep#1**	**Rep#2**	**Rep#3**
**WT**	32	16	32
**Δ*fsr***	32	16	32

Table S3. PCR screening to detect the presence (+) or absence (-) of *fsrA* in *E. faecalis* isolates from multiple sampling occasions per patient

	Sampling occasion						
**Patient ID**	**1**	**2**	**3**	**4**	**5**	**6**	**7**
ENVALVE_009	-						
ENVALVE_036	-	-	-				
ENVALVE_049	-						
ENVALVE_065	-	-					
ENVALVE_069	+						
ENVALVE_126	+						
ENVALVE_127	-						
ENVALVE_132	-						
ENVALVE_158	-						
ENVALVE_159	-	-	-	-	-		
ENVALVE_162	-	-					
ENVALVE_163	+						
ENVALVE_207	-	-	-				
ENVALVE_213	-	-	-				
ENVALVE_280	+	+	+	+	+		
ENVALVE_291	-	-	-	-	-	-	
ENVALVE_308	-	-	-				
ENVALVE_317	-	-	-	-	-	-	-
ENVALVE_350	-						
ENVALVE_358	+	+	+	+	+		
ENVALVE_364	-	-					
ENVALVE_391	-	-	-				
ENVALVE_395	+	+					
ENVALVE_396	+						

SUPPLEMENTARY INFORMATION UNCROPPED SCANS OF BLOTS

Gel lanes											
1	2	3	4	5	6	7	8	9	10	11	12
Ladder	WT	Δ*gelE*	Δ*gelE* pTCV	*gelE* pTCV-*gel*	BHI	Ladder	OTHER EXPERIMENT				

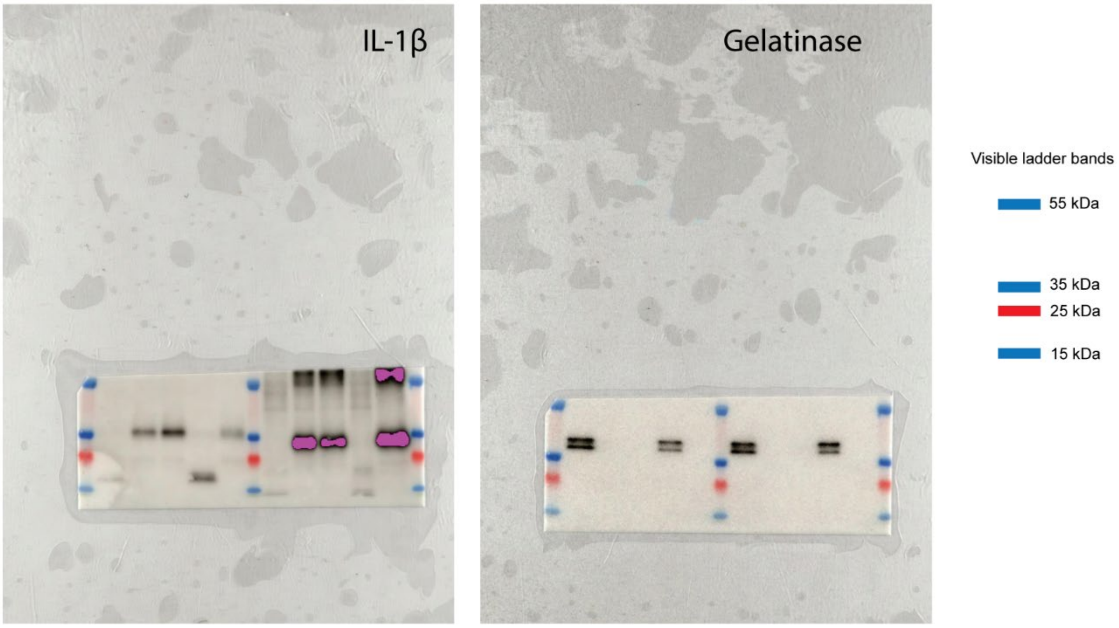

Uncropped scans for Fig. S4G. Green highlights the lanes shown in Fig. S4G.
