# Supplementary material for: Loss of Fsr quorum sensing promotes biofilm formation and worsens outcomes in enterococcal infective endocarditis
Source: Nat Commun. 2026 Jan 14;17:1668. doi: 10.1038/s41467-026-68366-8 (PMC12909850; doi:10.1038/s41467-026-68366-8)
Supplement: Supplementary file 2 — Description of Additional Supplementary File [file 41467_2026_68366_MOESM2_ESM.pdf]

**Description of Additional Supplementary Files**

**Supplementary Data 1:** Differential gene expression of *E. faecalis* under fluid flow compared to static conditions**.**

**Supplementary Data 2:** Differential gene expression of *E. faecalis* OG1RF Δ*fsr* compared to WT in vegetation at 72 hpi.

**Supplementary Data 3:** Baseline characteristics of 81 patients with enterococcal IE stratified by *fsrA* presence and cohort.

**Supplementary Data 4:** Human pro-IL-1β peptides identified by mass spectrometry after incubation with *E. faecalis* for 0, 6, and 18 h

**Supplementary Data 5:** *E. faecalis* strains and plasmids used in *in vitro* and *in vivo* assays**.**

**Supplementary Data 6:** Oligonucleotides used in this study**.**
