# Supplementary material for: Loss of Fsr quorum sensing promotes biofilm formation and worsens outcomes in enterococcal infective endocarditis
Source: Nat Commun. 2026 Jan 14;17:1668. doi: 10.1038/s41467-026-68366-8 (PMC12909850; doi:10.1038/s41467-026-68366-8)
Supplement: Supplementary file 9 — Reporting summary [file 41467_2026_68366_MOESM9_ESM.pdf]

nature portfolio

Corresponding author(s): Haris Antypas, Kimberly A. Kline

Last updated by author(s): Dec 12, 2025

Reporting Summary

Nature Portfolio wishes to improve the reproducibility of the work that we publish. This form provides structure for consistency and transparency in reporting. For further information on Nature Portfolio policies, see our Editorial Policies and the Editorial Policy Checklist.

Statistics

For all statistical analyses, confirm that the following items are present in the figure legend, table legend, main text, or Methods section.

n/a	Confirmed	
☐	☒	The exact sample size (*n*) for each experimental group/condition, given as a discrete number and unit of measurement
☐	☒	A statement on whether measurements were taken from distinct samples or whether the same sample was measured repeatedly
☐	☒	The statistical test(s) used AND whether they are one- or two-sided<br>*Only common tests should be described solely by name; describe more complex techniques in the Methods section.*
☐	☐	A description of all covariates tested
☐	☒	A description of any assumptions or corrections, such as tests of normality and adjustment for multiple comparisons
☐	☒	A full description of the statistical parameters including central tendency (e.g. means) or other basic estimates (e.g. regression coefficient) AND variation (e.g. standard deviation) or associated estimates of uncertainty (e.g. confidence intervals)
☐	☒	For null hypothesis testing, the test statistic (e.g. *F*, *t*, *r*) with confidence intervals, effect sizes, degrees of freedom and *P* value noted<br>*Give P values as exact values whenever suitable.*
☒	☐	For Bayesian analysis, information on the choice of priors and Markov chain Monte Carlo settings
☒	☐	For hierarchical and complex designs, identification of the appropriate level for tests and full reporting of outcomes
☒	☐	Estimates of effect sizes (e.g. Cohen's *d*, Pearson's *r*), indicating how they were calculated

Our web collection on statistics for biologists contains articles on many of the points above.

Software and code

Policy information about availability of computer code

Data collection	NA
Data analysis	Briefly, quality control of the RNA sequencing data was performed using FastQC, followed by filtering with SortMeRNA (Version 4.3.6) to remove rRNA sequences. Reads were aligned to the E. faecalis OG1RF reference genome (Accession number: GCF_000172575.2_ASM17257v2) using Bowtie2 (Version 2.5.3) 90 and reads per gene were quantified using HTSeq-count (Version 2.0.5). Differential expression analysis was conducted with edgeR (Version 3.36.0) with statistical significance set at FDR < 0.05. GO enrichment analysis was performed using goseq (Version 1.50.0).<br>Image analysis using ImageJ (version 1.54f)<br>Flow cytometry data were analyzed using FlowJo (version 10.7.1).<br>Statistical analysis for in vivo and in vitro assays was performed using GraphPad Prism 6 (version 6.07)

For manuscripts utilizing custom algorithms or software that are central to the research but not yet described in published literature, software must be made available to editors and reviewers. We strongly encourage code deposition in a community repository (e.g. GitHub). See the Nature Portfolio guidelines for submitting code & software for further information.

Data

Policy information about availability of data

All manuscripts must include a data availability statement. This statement should provide the following information, where applicable:

- Accession codes, unique identifiers, or web links for publicly available datasets
- A description of any restrictions on data availability
- For clinical datasets or third party data, please ensure that the statement adheres to our policy

Source data are provided with this paper. Transcriptomic data generated in this study have been deposited in NCBI under the bioproject accession numbers PRJNA1219810 and PRJNA1219807. Illumina data for the ENVALVE cohort isolates generated in this study have been deposited in NCBI under the bioproject accession number PRJNA1333350. MS proteomic data generated in this study have been deposited to MassIVE under accession number MSV000097326. GC-MS data generated in this study have been deposited in Yareta repository under DOI 10.26037/yareta:gsciqvezcffqpoxxie5qfy7nyy

Source data are provided with this paper, including original western blot images in this paper are provided in the Source Data File and Supplementary Information. This paper does not generate original codes.

Research involving human participants, their data, or biological material

Policy information about studies with human participants or human data. See also policy information about sex, gender (identity/presentation), and sexual orientation and race, ethnicity and racism.

Reporting on sex and gender	NA
Reporting on race, ethnicity, or other socially relevant groupings	NA
Population characteristics	Detailed in Supplementary File 3
Recruitment	Inclusion Criteria:<br><br>• All patients with IE or clinical high suspicion of endocarditis, if:<br><br>o Age >18 years<br><br>o Written informed consent by the participant after information about the research project<br><br>• If there is a written directive with the alleged will of the patient we will consider this at every moment<br><br>• In patients with transitory mental disorders or transitory impossibility to sign we will ask an independent physician not involved in the research project to represent the patient, and as soon as the patient can express his opinion, an informed consent will be obtained. If the patient denies we will destroy the blood samples.<br><br>• In patients with long standing impossibility to sign because of the health status we will get the alleged will of the patient asking the relatives or the legale spokeperson and/or legal guardian guardian and as soon as possible we will ask the patient for his/her consent. If the patient denies we will destroy the blood samples. . We will ask the corresponding person about the alleged will during a talk. An informed consent will be signed by the relatives/legal spokeperson and as soon as possible we will talk with the patient and ask for his/her signed informed consent.<br><br>• In the case of permanently incapacitated persons, we will ask to give their consent to the person authorized to act as substitute for the permanent representation (ZGB Art. 378). They will signed the informed consent<br><br>Exclusion Criteria:<br><br>• Contraindication on ethical grounds (Dementia, impossibility to understand because of language problems)
Ethics oversight	Clinical data and 24 E. faecalis isolates from IE patients were collected in accordance with the ethical guidelines of the independent ethics committee Zurich, Switzerland. The study was approved under BASEC ID 2017-01140 and 2017-02225. Informed consent was obtained from all participants prior to data collection. Previously published clinical data and genomic information from 57 E. faecalis isolates collected from patients with definite or probable IE at University of Pittsburgh Medical Center were also included 69. This study was approved with a waiver of informed consent by the Institutional Review Board at the University of Pittsburgh (protocol no. STUDY22050046).

Note that full information on the approval of the study protocol must also be provided in the manuscript.

Field-specific reporting

Please select the one below that is the best fit for your research. If you are not sure, read the appropriate sections before making your selection.

☒ Life sciences ☐ Behavioural & social sciences ☐ Ecological, evolutionary & environmental sciences

For a reference copy of the document with all sections, see nature.com/documents/nr-reporting-summary-flat.pdf

Life sciences study design

All studies must disclose on these points even when the disclosure is negative.

Sample size	For human samples: No sample size calculations were performed, as the samples were already collected and further used for this project. All E. faecalis invasive strains of IE patients available in the two studies were used for this project.<br>For animal studies: The sample size was determined based on previous studies in IE, ensuring that the number of animals used was sufficient to observe biologically relevant effects while minimizing unnecessary animal use.
Data exclusions	For human samples: One Patient was excluded whose invasive strain was analysed but no clinical data was available to properly analyse the clinical implications of his gene presence/absence.<br>For animal studies: Animals with incorrect catheter placement were excluded from downstream analysis.
Replication	For human samples: NA because there were no experimental findings<br>For animal studies: Findings were confirmed across independent animals and across separate breeding batches<br>In vitro experiments were performed in independent biological replicates performed on separate occasions. Technical replicates were includes to assess intra-assay variability.<br>All attempts at replication for both in vivo and in vitro were successful.
Randomization	For human samples: NA<br>For animal studies: Allocation of animals was random
Blinding	For human samples: NA<br>For animal/in vitro studies: With the exception of microscopy analysis, blinding was not implemented because data generated and analyzed were not subject to observer bias. To minimize bias in microscopy analysis, image acquisition and quantification in imajeJ were blinded.

Reporting for specific materials, systems and methods

We require information from authors about some types of materials, experimental systems and methods used in many studies. Here, indicate whether each material, system or method listed is relevant to your study. If you are not sure if a list item applies to your research, read the appropriate section before selecting a response.

Materials & experimental systems

n/a	Involved in the study	
☐	☒	Antibodies
☐	☒	Eukaryotic cell lines
☒	☐	Palaeontology and archaeology
☐	☒	Animals and other organisms
☒	☐	Clinical data
☒	☐	Dual use research of concern
☒	☐	Plants

Methods

n/a	Involved in the study	
☒	☐	ChIP-seq
☐	☒	Flow cytometry
☒	☐	MRI-based neuroimaging

Antibodies

Antibodies used	Antibodies and respective dilutions used for immunofluorescence (IF) and western blotting (WB) in this study include rabbit anti-Streptococcus Group D (Antigen D) (1:500 for IF, Cat. Nr. 12-6231D, American Research Products, Inc), mouse anti-myeloperoxidase (1:50 for IF, Cat. Nr. NBP1-51148, Novus Biologicals), rabbit anti-IL-1β (1:50 for IF and 1:1000 for WB, Cat. Nr. ab283818, abcam), rabbit anti-gelE (1:500 for WB, Cat. Nr. PA5-117682, Invitrogen), rabbit anti-beta-actin (1:4000 for WB, Cat. Nr. ab8227, abcam), goat anti-Rabbit IgG (H+L) HRP-conjugated antibody (1:5000 for WB, Cat. Nr. 31460, Invitrogen), goat anti-rabbit IgG Alexa 488 (1:1000 for IF, Cat. Nr. A11034, Invitrogen), goat anti-mouse IgG1 Alexa 633 (1:1000 for IF, Cat. Nr. A-21126, Invitrogen).
Validation	All antibodies are commercially available and validated for the applications used in this manuscript by the manufacturer or as reported by citations found in the manufacturer's website.

Eukaryotic cell lines

Policy information about cell lines and Sex and Gender in Research

Cell line source(s)	NA
Authentication	NA
Mycoplasma contamination	HEK-Blue IL1R cells (invivogen) used in this study tested negative for mycoplasma.
Commonly misidentified lines (See ICLAC register)	NA

Animals and other research organisms

Policy information about studies involving animals; ARRIVE guidelines recommended for reporting animal research, and Sex and Gender in Research

Laboratory animals	Rattus norvegicus Sprague Dawley, 7-9wo
Wild animals	NA
Reporting on sex	NA
Field-collected samples	NA
Ethics oversight	Rats were used in accordance with the NTU Institutional Animal Care and Use Committee guidelines (Animal utilization protocols A19091 and A24076).

Note that full information on the approval of the study protocol must also be provided in the manuscript.

Plants

Seed stocks	NA
Novel plant genotypes	NA
Authentication	NA

Flow Cytometry

Plots

Confirm that:

☒ The axis labels state the marker and fluorochrome used (e.g. CD4-FITC).

☒ The axis scales are clearly visible. Include numbers along axes only for bottom left plot of group (a 'group' is an analysis of identical markers).

☒ All plots are contour plots with outliers or pseudocolor plots.

☒ A numerical value for number of cells or percentage (with statistics) is provided.

Methodology

Sample preparation	Aortic valve vegetations were excised, weighed, and minced in 500 µL of digestion buffer (0.1 mg/mL Liberase in PBS). Samples were incubated at 37°C for 30 min with gentle rocking. After incubation, 500 µL of flow cytometry staining buffer (FCSB; 1 % BSA and 0.1% sodium azide in PBS) was added, and the supernatants were filtered through a 35-µm cell strainer into a 50 mL tube. An additional 500 µL of FCSB was used to flush the strainer, bringing the total volume to 1.5 mL. Filtrates were centrifuged at 500 × g for 5 min at 4°C, and cell pellets were resuspended in 200 µL of FCSB. 100 µL per sample were subsequently used for each staining condition. Non-specific Fc receptor binding was blocked by incubating with mouse anti-CD32 (1:100, Cat. Nr. 550270, BD Pharmingen) for 20 min at 4°C. Following blocking, samples were incubated with mouse anti-CD45:Alexa Fluor 700 (1:10, Cat. Nr. MCA43A700, Biorad) and mouse anti-RP-1:Alexa Fluor 647 (1:20, Cat. Nr. 550000, BD Pharmingen) for 30 min at 4°C in the dark. After incubation, cells were washed and fixed in 4 % PFA for 15 min at 4°C in

the dark, followed by washing and resuspension in 100 µL FCSB. To obtain absolute cell counts, 100 µL of AccuCheck Counting Beads (Thermo Fisher Scientific) were added to each sample by reverse pipetting. Stained cells were acquired using a BD LSRFortessa X-20 flow cytometer equipped with 5 lasers (488nm, 535nm, 633nm, 405nm, 355nm). A total of 10000 events were collected per sample. Compensation was applied using the AbC Anti-Mouse Bead Kit (Thermo Fisher Scientific), and fluorescence-minus-one (FMO) controls were used to set gating thresholds. Flow cytometry data were analyzed using FlowJo (version 10.7.1). Absolute cell counts were calculated according to the manufacturer's instructions for AccuCheck Counting Beads and normalized to total vegetation weight.

Instrument	BD LSRFortessa X-20 flow cytometer equipped with 5 lasers (488nm, 535nm, 633nm, 405nm, 355nm)
Software	Flow cytometry data were analyzed using FlowJo (version 10.7.1).
Cell population abundance	No cell sorting performed
Gating strategy	Fluorescence-minus-one (FMO) controls were used to set gating thresholds. See Fig. S10.

☒ Tick this box to confirm that a figure exemplifying the gating strategy is provided in the Supplementary Information.
