# Supplementary material for: Author Correction: Loss of Fsr quorum sensing promotes biofilm formation and worsens outcomes in enterococcal infective endocarditis
Source: Nat Commun. 2026 Sep 25;17:10214. doi: 10.1038/s41467-026-78152-1 (PMC13612818; doi:10.1038/s41467-026-78152-1)
Supplement: Supplementary file 1 [file 41467_2026_78152_MOESM1_ESM.pdf]

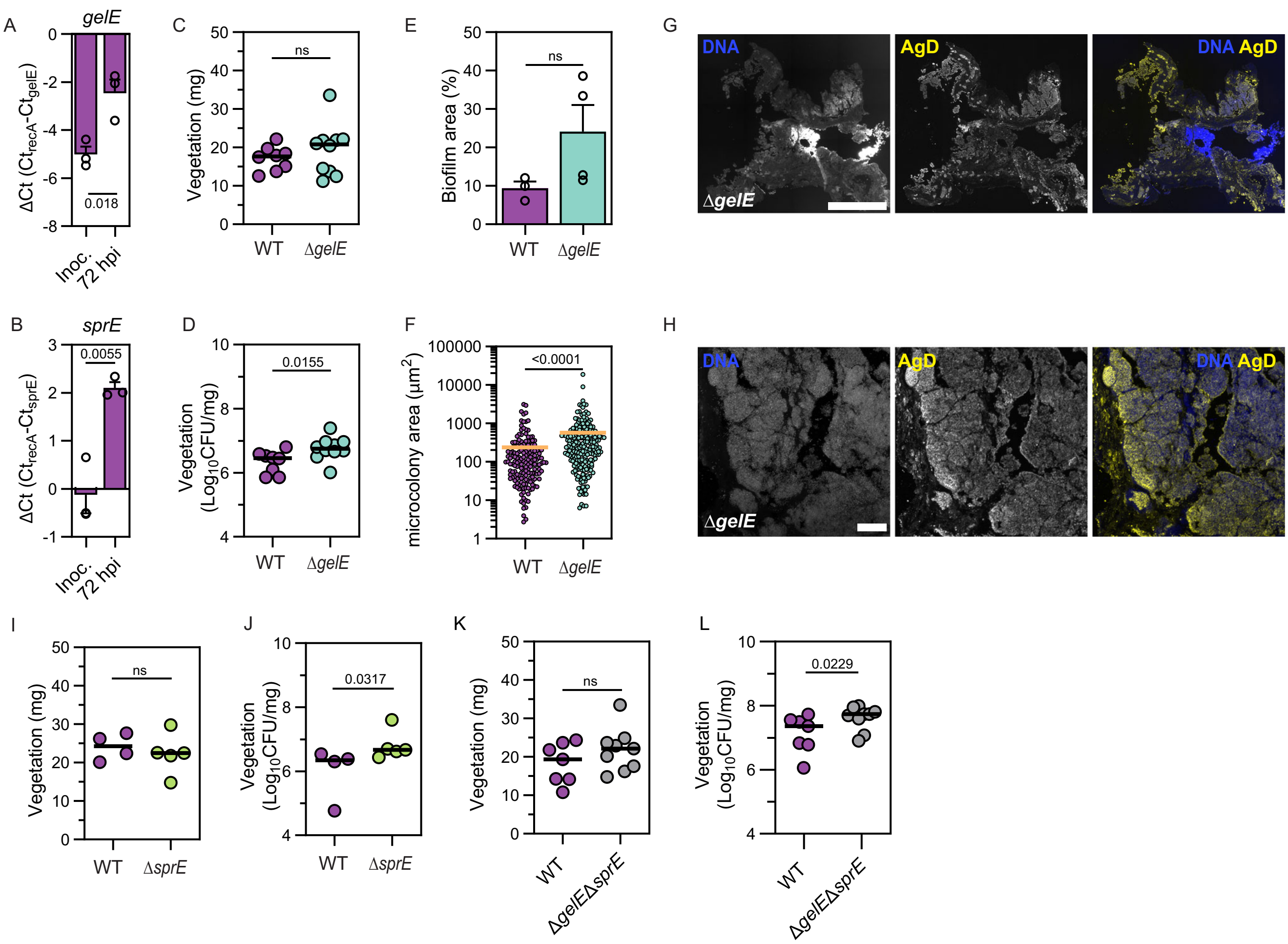

Fig. 4 | Absence of *gelE* and *sprE* contributes to biofilm growth in late IE. **A**, **B** qPCR analysis showing mean *gelE* and *sprE* expression of the WT strain in the inoculum and vegetations at 72 hpi. *gelE* expression is presented as ΔCt values normalized to the expression of *recA*. Vegetation cDNA samples derived from n = 3, N = 2. Error = SEM. Statistical significance was determined using a two-tailed sample t-test. **C**, **D** Vegetation weight (**C**) and CFU (**D**) at 72 hpi. Median shown for *n* = 8 (WT) and *n* = 10 (Δ*gelE*), N = 2. Statistical significance assessed with a two-tailed Mann-Whitney test. **E** Mean biofilm area (%) per vegetation section shown from *n* = 4, N = 2. Biofilm area was determined by calculating the antigen D positive area to total vegetation area. Error = SEM. Statistical significance was assessed with t-test. **F** Distribution and median of intravegetational microcolony area shown from *n* = 3 (WT) and *n* = 4 (Δ*gelE*), N = 2. A total of 168 microcolonies for WT and 221 microcolonies for Δ*gelE* were quantified. Statistical significance was determined using a two-tailed Mann-Whitney test. **G** Epifluorescence microscopy tile images of whole vegetation sections from 72 hpi, stained for DNA and antigen D (AgD). Representative section shown from *n* = 4, N = 2. Scale = 500 μm. **H** Z-projection of biofilm microcolonies from 72 hpi captured with LSCM stained for DNA and AgD. Representative image shown from n = 4, N = 2. Scale = 20 μm. **I**–**L** Vegetation weight and CFU at 72 hpi. Median of *n* = 4 (WT) and *n* = 5 (Δ*sprE*), N = 1 for (**I**) and (**J**), and *n* = 7 (WT) and *n* = 9 (Δ*gelE*Δ*sprE*), N = 2 for (**K**) and (**L**) is shown, with statistical significance assessed with a two-tailed Mann-Whitney test; *ns* not significant ($p \geq 0.05$), *n* = animals per group, N = independent experiments. Exact p-values are reported in the figure. Source data are provided as a Source Data file.
